# DNA Vaccine-Generated Duck Polyclonal Antibodies as a Postexposure Prophylactic to Prevent Hantavirus Pulmonary Syndrome (HPS)

**DOI:** 10.1371/journal.pone.0035996

**Published:** 2012-04-27

**Authors:** Rebecca Brocato, Matthew Josleyn, John Ballantyne, Pablo Vial, Jay W. Hooper

**Affiliations:** 1 Virology Division, United States Army Medical Research Institute of Infectious Diseases, Fort Detrick, Maryland, United States of America; 2 Aldevron, LLC, Fargo, North Dakota, United States of America; 3 Institute of Science, Medical School, Clinica Alemana Universidad del Desarrollo, Santiago, Chile; University of Texas Medical Branch, United States of America

## Abstract

Andes virus (ANDV) is the predominant cause of hantavirus pulmonary syndrome (HPS) in South America and the only hantavirus known to be transmitted person-to-person. There are no vaccines, prophylactics, or therapeutics to prevent or treat this highly pathogenic disease (case-fatality 35–40%). Infection of Syrian hamsters with ANDV results in a disease that closely mimics human HPS in incubation time, symptoms of respiratory distress, and disease pathology. Here, we evaluated the feasibility of two postexposure prophylaxis strategies in the ANDV/hamster lethal disease model. First, we evaluated a natural product, human polyclonal antibody, obtained as fresh frozen plasma (FFP) from a HPS survivor. Second, we used DNA vaccine technology to manufacture a polyclonal immunoglobulin-based product that could be purified from the eggs of vaccinated ducks (*Anas platyrhynchos*). The natural “despeciation" of the duck IgY (*i.e.*, Fc removed) results in an immunoglobulin predicted to be minimally reactogenic in humans. Administration of ≥5,000 neutralizing antibody units (NAU)/kg of FFP-protected hamsters from lethal disease when given up to 8 days after intranasal ANDV challenge. IgY/IgYΔFc antibodies purified from the eggs of DNA-vaccinated ducks effectively neutralized ANDV *in vitro* as measured by plaque reduction neutralization tests (PRNT). Administration of 12,000 NAU/kg of duck egg-derived IgY/IgYΔFc protected hamsters when administered up to 8 days after intranasal challenge and 5 days after intramuscular challenge. These experiments demonstrate that convalescent FFP shows promise as a postexposure HPS prophylactic. Moreover, these data demonstrate the feasibility of using DNA vaccine technology coupled with the duck/egg system to manufacture a product that could supplement or replace FFP. The DNA vaccine-duck/egg system can be scaled as needed and obviates the necessity of using limited blood products obtained from a small number of HPS survivors. This is the first report demonstrating the *in vivo* efficacy of any antiviral product produced using DNA vaccine-duck/egg system.

## Introduction

Andes virus (ANDV) is responsible for the majority of hantavirus pulmonary syndrome (HPS) cases in the South American countries of Argentina, Brazil, Chile, and Uruguay [1]. Between 1995–2008, over 700 reported cases of HPS in Argentina alone [2], 680 in Chile (1995–2010) [3], and 884 in Brazil (1993–2007) [4] with more cases throughout South, Central, and North America. Infection is thought to occur primarily through inhalation or ingestion of rodent excreta, or by rodent bites. However, there is convincing evidence that ANDV can be transmitted from person-to-person, resulting in clusters of cases [5], [6]. The case-fatality-rate for HPS is approximately 40% and there are currently no licensed vaccines, therapeutics, or postexposure prophylactics for this disease [7]. Efforts to develop medical countermeasures to prevent and treat HPS have been bolstered by the use of the ANDV/Syrian hamster model of lethal HPS. This model accurately mimics human HPS disease in incubation time, tropism to endothelial cells, thrombocytopenia, neutrophilia, lung pathology including pulmonary edema and pleural effusion, and shock [8], [9], [10], [11], [12], [13]. The ANDV/Syrian hamster model has been used to evaluate proof-of-concept vaccines [14], [15] and postexposure prophylactics [14], [16].

Historically, one of the most effective approaches to prevent and treat persons exposed to pathogenic viruses has been the use of antiserum. For example, persons potentially exposed to rabies virus are administered rabies antiserum, and are then vaccinated. Similarly, antiserum has been used to successfully treat Argentinean hemorrhagic fever [17], [18]. Passive vaccination to prevent hantavirus disease was previously investigated in our laboratory. We demonstrated that plasma from a HPS survivor was sufficient to protect in the ANDV/hamster model [14]. We also found that serum containing neutralizing antibodies collected from rhesus macaques or rabbits vaccinated with a DNA vaccine containing the M segment of ANDV (pWRG/AND-M) protected hamsters from lethal disease after intramuscular challenge with ANDV up to 5 days postchallenge [16]. These studies clearly demonstrated that passive protection using nonpurified polyclonal antibodies collected from survivors, or produced using DNA vaccine technology, can be an effective approach to preventing hantavirus disease even when administered days after exposure.

Despite the promising role of antibodies as ANDV immunotherapeutics, there are no neutralizing monoclonal antibodies and human convalescent sera are very rare. While our previous work using sera from nonhuman primates and rabbits suggests using antibodies from these animals may be a viable option, the risks of reactogenicity, including serum sickness, are high [19]. A possible solution is the use of duck-generated antibodies. Ducks produce three immunoglobulin isotypes, IgM, IgA, and IgY. Expression of the IgY isotype can be alternatively spliced creating an IgY lacking the Fc region (IgYΔFc) in hypervaccinated ducks [20], [21]. Because the Fc region is predominantly responsible for reactogenicity [22], a truncated isoform is an attractive option when neutralization is the primary goal. Ducks have been vaccinated with purified detoxified venom antigens from various snakes, and the IgYΔFc purified from egg yolks and tested in the development of antitoxins [23]. This strategy has been evaluated in a hepadnavirus infection model. In that study, ducks were vaccinated with a DNA vaccine encoding hepadnavirus envelope proteins. The eggs from the ducks contained IgYΔFc and ducklings produced by the vaccinated ducks were protected against hepadnavirus challenge [24], [25]. This approach has also been evaluated in a mouse influenza model where IgY from vaccinated laying chickens protects mice from lethal highly pathogenic avian influenza [26].

Here, we used human polyclonal antibodies (i.e., fresh frozen plasma from an HPS survivor) to define the dose in neutralizing units required to protect, and the pre-disease onset timeframe required for effective treatment. In addition, we explored the concept of manufacturing antiviral neutralizing polyclonal antibodies in ducks using DNA vaccine technology, purifying the candidate product from duck egg yolks, and testing the material in an animal model of lethal HPS. We demonstrate, for the first time, that it is possible to manufacture a polyclonal postexposure prophylactic product that targets a lethal viral disease using a combination of DNA vaccine and duck egg technology.

## Materials and Methods

### Ethics statement

Research was conducted in compliance with the Animal Welfare Act and other federal statutes and regulations relating to animals and experiments involving animals and adheres to principles stated in the Guide for the Care and Use of Laboratory Animals, National Research Council, 1996. The facilities where this research was conducted are fully accredited by the Association for Assessment and Accreditation of Laboratory Animal Care International. All animal experiments were approved by USAMRIID's Institutional Animal Care and Use Committee (approval ID AP-08-008). Human plasma was collected with written informed consent in accordance with an NIH sponsored clinical protocol approved by the Comité de Etica de la Investigación, Centro De Bioética Facultad de Medicina , Clínica Alemana, Universidad del Desarrollo (funded by NIH U01AI045452). Animal work involving passive transfer of human plasma was approved by the USAMRIID Human Use Committee, under Exemption Certificate FY07-20, HP-07-20.

### Virus and cells

A twice plaque-purified ANDV strain Chile-9717869 passaged two times in Vero E6 cells (Vero C1008 ATCC CRL 1586, Manassas, VA) was described previously [27]. Cells were maintained in Eagle's minimal essential medium with Earle's salts (EMEM) supplemented with 10% fetal bovine serum (FBS), 10 nM HEPES (pH 7.4), 200 U/ml penicillin, 200 µg/ml streptomycin, 1× nonessential amino acids (NEAA), 1.5 µg/ml amphotericin B, and 50 µg/ml gentamicin sulfate (cEMEM) at 37°C in a 5% CO_2_ incubator.

### Anti-ANDV FFP

Fresh frozen plasma (FFP) was obtained, with informed consent, from a convalescent HPS patient infected with ANDV. Typically, FFP is frozen within 8 h of collection at a temperature of at least −20°C. Before use in passive transfer experiments, α-ANDV FFP and normal FFP were thawed on wet ice, heat inactivated (56°C, 30 min) and aliquoted.

### PRNT

Plaque reduction neutralization tests (PRNT) were performed as previously described [11]. Briefly, heat-inactivated serum samples (56°C for 30 min) were diluted in cEMEM. Samples containing purified IgY/IgYΔFc were not heat-inactivated. These dilutions were mixed with an equal volume of approximately 75 PFU of ANDV with or without guinea pig or human complement at a final concentration of 5% (Accurate Chemical and Scientific Corp., Westbury, NY). This mixture was incubated overnight at 4°C and then a plaque assay was performed as described by using 7-day old Vero E6 monolayers in 6-well plates. After 7 days, monolayers were stained with neutral red (Invitrogen, Carlsbad, CA) and plaques were counted 2 days (37°C) after staining. Complement has been reported to enhance hantavirus neutralizing antibody titers for certain samples, and consequently our standard PRNT includes complement [11], [16], [27]. PRNT titers represent the highest serum dilution which neutralizes 80% of the plaques in control (no serum) wells. Neutralizing antibody units (NAU) are the PRNT_80_ value per ml. For example, a sample with a PRNT_80_ value of 2560 would have 2560 NAU/ml.

### Intranasal and intramuscular injection of hamsters with virus

Female Syrian hamsters (*Mesocricetus auratus*) weighing 150 to 200 g (Harlan, Indianapolis, IN) were anesthetized by an intramuscular (i.m.) injection with approximately 0.1 ml/100 g of body weight of ketamine-acepromazine-zylazine mixture. For intranasal (i.n.) injections, anesthetized hamsters were administered 50 µl delivered as 25 µl per nare with a plastic pipette tip (4,000 PFU ANDV total, 42 LD_50_). Intramuscular (i.m.) injections in the caudal thigh consisted of 2,000 PFU (250 LD_50_) or 200 (25 LD_50_) of ANDV diluted in PBS to a volume of 0.2 ml. After viral challenge, hamsters were placed in isolator units (one to four hamsters per cage). Groups of 8 hamsters were typically used for experimental treatments, unless otherwise stated. All work involving hamsters was performed in an animal biosafety level 4 (ABSL-4) laboratory. Hamsters were observed two to three times daily. Subcutaneous [17] injections of test article were conducted in the scruff of the neck with 0.5–1 ml volume.

### Vaccination of ducks

Khaki Campbell ducks (*Anas platyrhynchos*) were received as hatchlings and raised in a facility isolated from all other species. Ducks were vaccinated at approximately 7 months of age (average weight 1.3 kg) by mildly anesthetizing with isoflurane before each electroporation event. Ducks were initially vaccinated with a priming dose of 320 µg pWRG/AND-M delivered over two sites in the breast muscle with a twin injection device (Inovio, Blue Bell, PA). Thereafter, 160 µg of the plasmid was delivered bilaterally on days 14, 28, 56, 70, 84, and 252. Nonresponsive ducks, as measured by PRNT, were removed from the study while remaining ducks were vaccinated with 1 mg total dose administered bi-laterally with the TriGrid device (Ichor Medical Systems, San Diego, CA) on days 483, 504, 525, 553, and 588. Duck eggs were collected and cracked and the contents gently transferred to a yolk separator to allow a majority of the egg white to separate by gravity from the yolk. A razor blade was used to cut the egg white on the outside of the yolk separator taking care not to damage the yolk sac. The yolk sac was then transferred to a paper towel and gently rolled to allow any remaining egg white to adhere. The yolk sac, typically 20 cc per egg, was transferred to a 2 L bottle. This procedure was repeated until the desired number of duck eggs had been pooled.

### Duck IgY/IgYΔFc purification from eggs

The egg yolks were diluted 10-fold in water and the pH of the mixture was adjusted to 5.0 with HCl. The diluted yolk suspension was agitated gently for 4 h at 4°C then centrifuged at 10,000× g for 30 min at 4°C. The semi-clarified supernatant was mixed with Celpure® P300 (Advanced Minerals Corporation, Goleta, CA) to a final concentration of 10% w/v by rolling the bottles and was then filtered through pre-caked GF/A filters (Whatman, Piscataway, NJ). The resulting filtrate then undertook 10 diavolumes of exchange into 10 mM Tris, pH 7.0 on a Pellicon II PES 100 kD MWCO (Millipore, Billerica, MA) tangential flow filter. Solid PEG 6000 (Fluka, Fuchs, Switzerland) was added to the solution to a final concentration of 20% w/v and was allowed to dissolve by mixing at room temperature for 30 min. The solution was then centrifuged at 10,000× g for 30 min at 4°C. After centrifugation the supernatant solution was discarded and the resulting pellet was dissolved in 50 mM Tris-HCl, pH 9.0 (Sigma-Aldrich, St. Louis, MO). A Fractogel TMAE (trimethylaminoethyl) (EMD Chemicals, Gibbstown, NJ) column was equilibrated with five bed volumes of 50 mM Tris-HCl, pH 9.0. The dissolved PEG fractionation pellet was then loaded onto the column and the load flow through was discarded. The column was washed with twenty bed volumes of 50 mM Tris-HCl to remove residual PEG 6000. The IgY was eluted from the column with four bed volumes of 50 mM Tris-HCl, pH 9.0 containing 500 mM NaCl. The IgY rich fraction was then exchanged with 12 diavolumes of phosphate buffered saline (pH 7.4). The concentration of the purified IgY/IgYΔFc was determined with a BSA standard based assay (Bio-Rad, Hercules, CA) and 280 nm absorbance relative to known IgY standards (Gallus).

### Western Blot Analysisz

IgY/IgYΔFc samples were separated in a 4–12% SDS-PAGE run under nonreducing conditions and blotted onto a PVDF membrane (Invitrogen, Carlsbad, CA). IgY, IgYΔFc, and antibody components were detected with a mouse monoclonal antibody to duck IgY, clone 16C7 (Thermo Fisher Scientific, Waltham, MA). Signals were visualized using chemiluminescence SuperSignal West Femto Substrate kit according to the manufacturer's instructions (Thermo Fisher Scientific, Waltham, MA). Signals were captured using GeneSnap software and a G:Box Imaging system (Syngene, Frederick, MD). Signal intensities were analyzed using GeneTools (Syngene, Frederick, MD).

### ELISA

Antibodies to the ANDV nucleocapsid (N) protein cross-react with purified Puumala virus N in an established ELISA [13], [28]. This Puumala N-based ELISA was used to detect evidence of ANDV infection in hamsters exposed to ANDV as described previously. All hamster serum samples were gamma-irradiated (on dry ice) with 3×10^6^ rads from a ^60^Cobalt source and then heat-inactivated (56°C, 30 minutes) before being serial-diluted (10-fold dilutions starting at 1∶100) and evaluated in the ELISA. Specific O.D. values are obtained by subtracting values from the sera sample using a negative control his-tagged protein as the solid phase antigen. Endpoint titers are defined as the highest dilution that produces a specific O.D. value>than mean of three negative control sera plus three standard deviations.

### Statistical Analysis

Half-life was calculated by nonlinear regression analysis using a sigmoidal dose response with variable slope. Survival data ere analyzed using Fisher's exact test (two-tailed). Mean day-to-death was analyzed using Mann-Whitney *U* test (two-tailed). Differences between antibody titers were analyzed using the student's t-test (two-tailed). *P* values of less than 0.05 were considered significant. All statistical analyses were performed using GraphPad Prism (La Jolla, CA) version 4.

## Results

### Neutralizing activity and bioavailability of anti-ANDV FFP used in hamster passive transfer experiments

**Figure 1:**
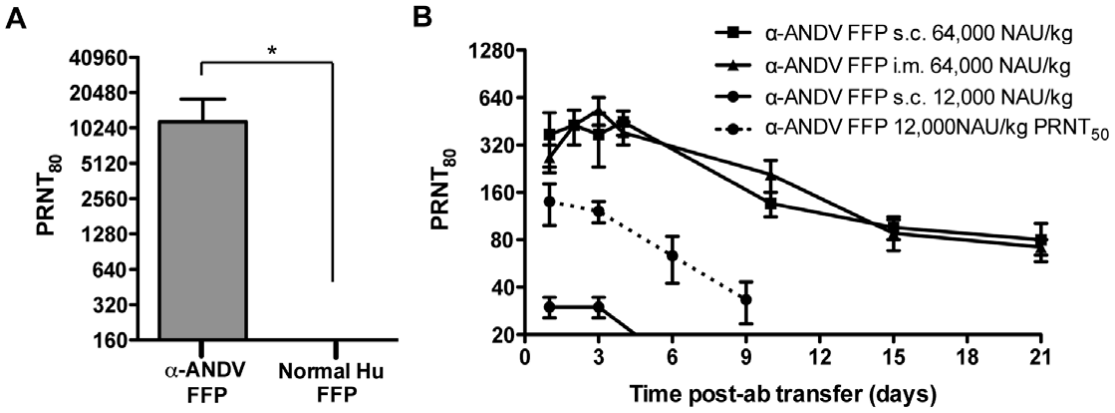
α-ANDV FFP effectively neutralizes ANDV *in vitro* and is detectable in hamsters after passive transfer. A) Neutralizing antibody titers were determined by ANDV PRNT_80_ performed on α-ANDV FFP and normal human serum. * indicates results are statistically significant. B) Neutralizing antibody bioavailability was determined by ANDV PRNT_80_ performed on hamster serum samples collected after passive transfer of α-ANDV FFP (64,000 NAU/kg) by either s.c. or i.m. route (3 hamsters per group) on day 0, through 21 days. PRNT_80_ titers represent the lowest serum dilution neutralizing 80% of the plaques relative to the control (no serum). PRNT_50_ values of a single group are denoted by a dashed line.

FFP from a Chilean HPS survivor and normal human FFP were tested for a capacity to neutralize ANDV by PRNT. Four independent PRNT_80_ were performed. The geometric mean titer (GMT) for the α-ANDV FFP was calculated to be 10,240 (**Fig. 1A** **,** **Table 1**). Neutralizing antibodies were not detected in normal human FFP.

**Table 1:** α-ANDV FFP and α-ANDV duck IgY/IgYΔFc stocks used per experiment.

Figure	α-ANDV Stock^a^	Concentration (mg/ml)	Dosage (mg/100 g hamster)	PRNT_80_	Neutralizing antibody dosage (NAU/kg)	Neutralizing antibody units (NAU/ml)
1	FFP	N/A	N/A	10240	64000	10240
2	FFP	N/A	N/A	10240	30720	10240
3	FFP	N/A	N/A	10240	30720, 5120,2560	10240
5,6	FFP	N/A	N/A	10240	12000	10240
4D	IgY/IgYΔFc Lot #1	11.4	4.56	1280	5000	1280
4B,5	IgY/IgYΔFc Lot #2	11.1	1.30	10240	12000	10240
6	IgY/IgYΔFc Lot #3	15.5	7.25	2560	12000	2560
4A, 4C	IgY/IgYΔFc Lot #4	12.8	6.00	2560	12000	2560
2	Rabbit Sera	N/A	N/A	640	1920	640

a FFP, fresh frozen plasma or duck IgYΔFc purified from vaccinated duck eggs.

Before performing passive transfer protection experiments, we measured the bioavailability of human neutralizing antibodies in hamsters injected with α-ANDV FFP. Groups of three animals were injected with either a high dose (64,000 NAU/kg) of α-ANDV FFP by either s.c. or i.m. routes or a lower dose (12,000 NAU/kg) of α-ANDV FFP by the s.c. route. Serum samples were obtained on days 1, 2, 3, 4, 10, 15, and 21, and neutralizing antibody titers were determined by PRNT (**Fig. 1B**). There was no statistical difference in the levels of neutralizing antibodies detected in hamster sera when α-ANDV FFP was administered at 64,000 NAU/kg by either s.c. or i.m. routes. Half-lives in hamster sera were calculated to be 7.7 days and 6.8 days when α-ANDV FFP was administered s.c. or i.m., respectively. Neutralizing antibodies were still detected on the last time-point tested (day 21). α-ANDV FFP administered at 12,000 NAU/kg s.c. was detectable for 3 days, then dropped below level of detection for a PRNT_80_. Plotting PRNT_50_ titers shows α-ANDV FFP is detectable out to 9 days. The half-lives of α-ANDV FFP administered at 12,000 NAU/kg are 4.2 days according to PRNT_80_ titers and 4.7 days according to PRNT_50_ titers. All of the protection experiments described below involved s.c. passive transfer.

### α-ANDV FFP effectively protects hamsters from lethal ANDV challenge

**Figure 2:**
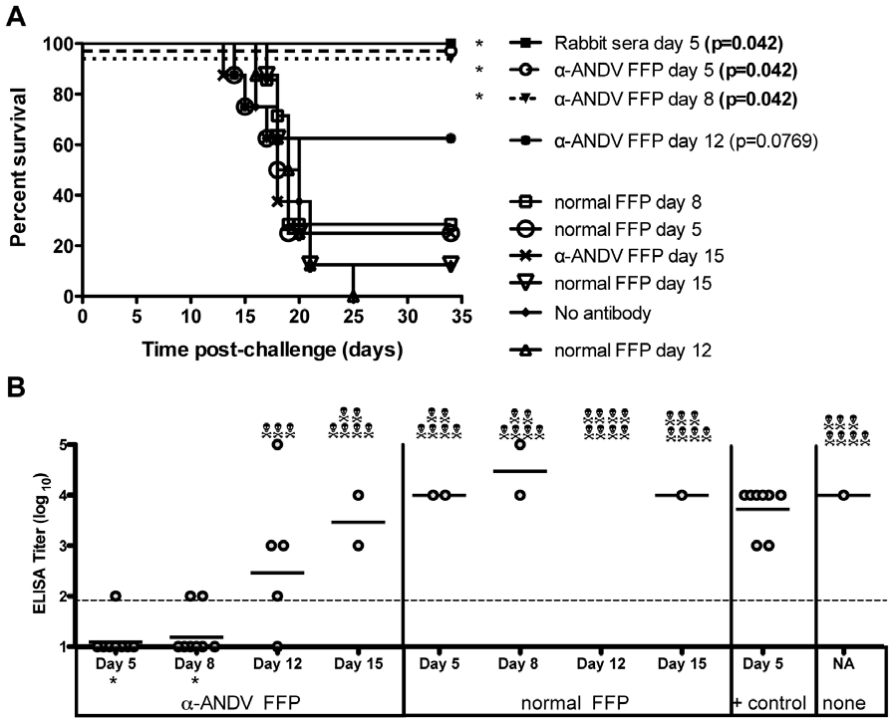
α-ANDV FFP passively transferred on days 5 and 8 protects hamsters from lethal disease and infection. A) Survival curve of hamsters challenged with 4,000 PFU i.n. of ANDV on day 0, then passively transferred with α-ANDV FFP (30,720 NAU/kg) on days 5, 8, 12 or 15 postinfection. Rabbit sera (administered at 1,920 NAU/kg) were collected 102 days post DNA vaccination [16]. P-values were determined based on comparison to normal serum on matching day. B) α-N ELISA endpoint titers (log_10_) were conducted with sera from surviving hamsters challenged with ANDV in A). GMT for each group are shown. * indicates results are statistically significant when compared to rabbit sera positive control.

We next determined the protective efficacy of α-ANDV FFP when administered at time-points post-ANDV challenge. Hamsters were challenged with 4,000 PFU of ANDV i.n. on day 0 and then received a single injection of 30,720 NAU/kg of α-ANDV, or normal FFP, on either day 5, 8, 12, or 15 after ANDV challenge. α-ANDV FFP administered on day 5 or 8 postchallenge was 100% effective at preventing lethal HPS disease in hamsters as determined by survival analysis. α-ANDV FFP administered on day 12 was 63% efficacious; however, this protection was not statistically significant in comparison to normal FFP (*P* = 0.0769) (**Fig 2A**).

To determine if surviving hamsters had been productively infected, sera from Figure 2 were subjected to a hantavirus nucleocapsid ELISA. Unexpectedly, hamsters receiving α-ANDV FFP on days 5 or 8 postchallenge had undetectable levels of α-nucleocapsid antibodies (**Fig. 2B**). These levels increased in surviving hamsters receiving α-ANDV FFP on days 12 and 15. By comparison, hamsters that received positive control α-ANDV rabbit sera (1,920 NAU/kg) on day 5 postchallenge had similar levels of antibodies compared to surviving hamsters that had received normal FFP or no antibody treatment. These data indicate that passive transfer of high levels of neutralizing antibodies can not only protect against lethal disease, but also can contain the infection in a manner that actually limits or, in some cases, prevents seroconversion (*P* = 0.0004 on α-ANDV FFP day 5 and *P* = 0.0005 on α-ANDV FFP day 8). Furthermore, these data demonstrate that even very high levels of neutralizing antibodies cannot protect against an i.n. challenge when administered on or after day 12, 5 days before the mean-day-death (day 17).

### Titration of anti-ANDV FFP protective dose in hamsters

**Figure 3:**
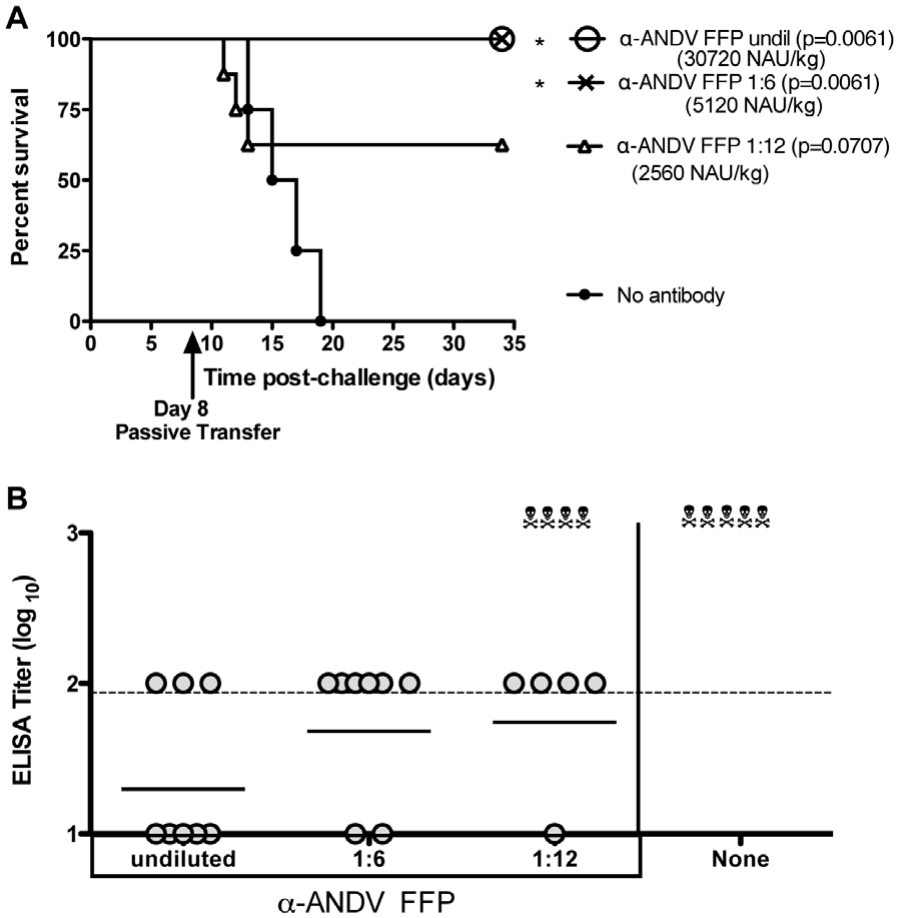
5,000 NAU/kg of α-ANDV FFP is sufficient to protect hamsters from lethal HPS disease. A) Survival curve of hamsters challenged with 4,000 PFU of ANDV i.n. on day 0 and passively transferred with dilutions of α-ANDV FFP on day 8. P-values were determined by comparing FFP dilution to no antibody control. B) α-N ELISA endpoint titers (log_10_) were conducted with sera from surviving hamsters challenged with ANDV in A). GMT for each group are shown.

Based on the results from the previous experiment showing undiluted α-ANDV FFP protected hamsters from lethal HPS, we next titrated the minimal dose of α-ANDV FFP required to elicit protection when administered post-ANDV challenge (4,000 PFU i.n.). Undiluted α-ANDV FFP (30,720 NAU/kg) along with 1∶6 and 1∶12 dilutions of α-ANDV FFP (corresponding to 5,120 and 2,560 NAU/kg) were passively transferred to ANDV-infected hamsters on day 8 postchallenge. Day 8 was selected based on the results in Figure 2 demonstrating that day 8 was the latest time-point that resulted in significant protection when a high dose of α-ANDV FFP was administered. Both the undiluted and the 1∶6 dilutions of α-ANDV FFP uniformly protected hamsters from ANDV-induced lethal HPS disease (**Fig. 3A**). Hamsters receiving the 1∶12 dilution of α-ANDV FFP had 63% survival, which was not statistically significant (*P* = 0.0707).

To determine if survivors had been productively infected, sera from surviving hamsters were subjected to nucleocapsid ELISA. Similar to the ELISA results in Figure 2B, hamsters receiving high dosages of α-ANDV FFP had low levels of α-ANDV antibodies (**Fig. 3B**). Of the hamsters receiving undiluted α-ANDV FFP, 5 of the 8 failed to develop a detectable immune response titers ≥2 (log 10). Of the hamsters receiving the 1∶6 or 1∶12 dilution, 75–80%, respectively, generated detectable humoral immune responses (*P* = 0.0005 for undiluted, *P* = 0.0005 for 1∶6, and *P* = 0.0023 for 1∶12, when compared to rabbit sera positive controls). Together, these data indicate that a single dose of α-ANDV FFP at ≥5,000 NAU/kg 8 days after exposure (9 days before mean day-to-death, 8 days prior to symptom onset) was sufficient to confer significant protection against lethal HPS. Moreover, higher doses of neutralizing antibodies not only protected against lethal disease, but also protected against productive infection as measured by seroconversion.

### A 12,000 NAU/kg dose of α-ANDV duck IgY/IgYΔFc is sufficient to protect hamsters from lethal ANDV i.n. challenge

**Figure 4:**
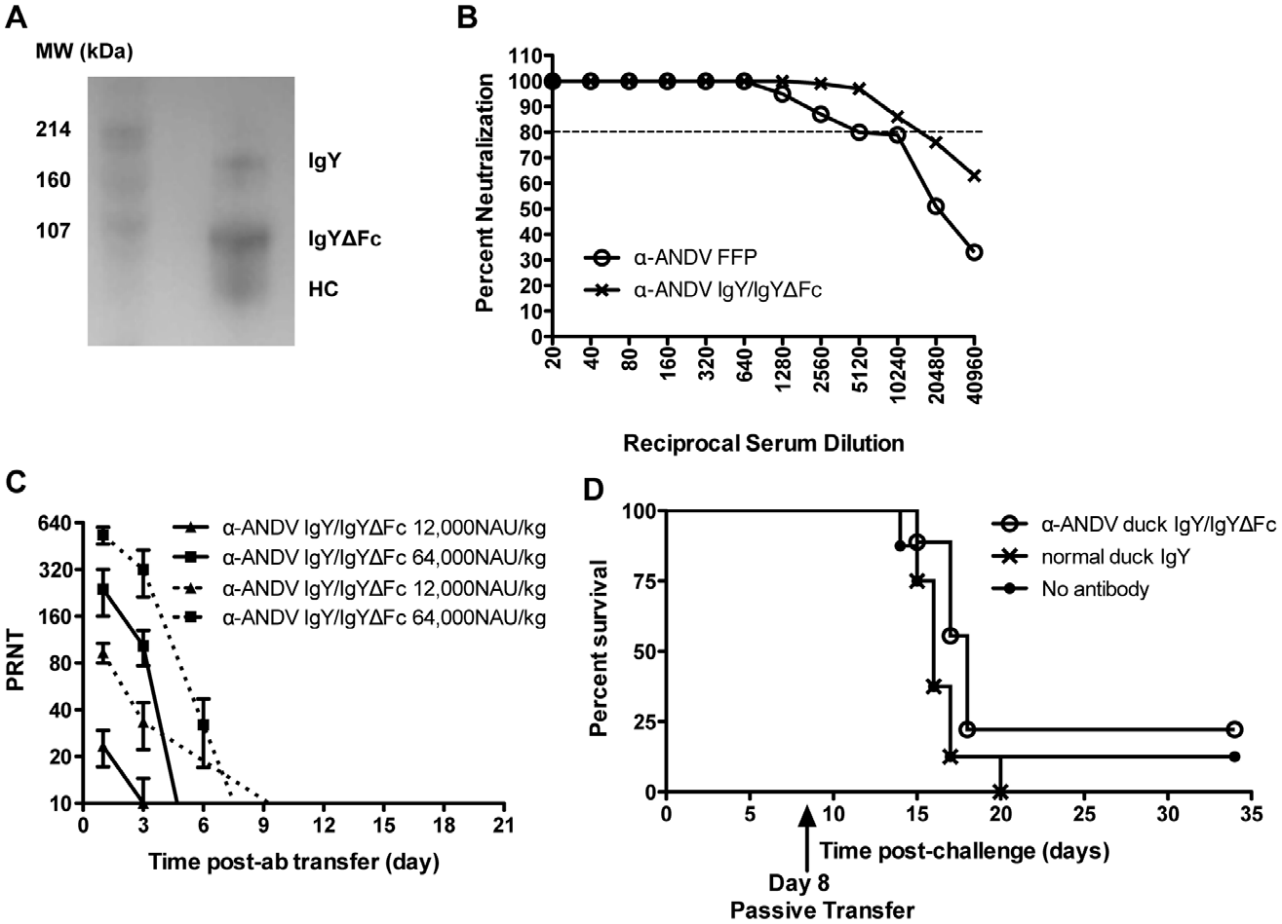
Neutralizing activity of α-ANDV duck IgY/IgYΔFc *in vitro* and *in vivo*. A) Western blot analysis of IgY components recognized by α-duck IgY antibodies. A) represents IgY/IgYΔFc by Western blot with SDS-PAGE run under non-reducing conditions and probed with an α-duck IgY antibody recognizing the heavy chain of both IgY and IgYΔFc. HC is the heavy chain of IgY. B) Percent neutralization of α-ANDV FFP and α-ANDV duck IgYΔFc measured by ANDV PRNT. Dotted line represents 80% neutralization (PRNT). C) Neutralizing antibody bioavailability was determined by ANDV PRNT performed on hamster serum samples collected after passive transfer of α-ANDV duck IgYΔFc (12,000 NAU/kg and 64,000 NAU/kg) by the s.c. route on day 0, through 21 days. PRNT_80_ titers (solid lines) and PRNT_50_ titers (dashed lines) are plotted. D) Survival curve of hamsters challenged with 4,000 PFU of ANDV i.n. on day 0 and passively transferred with 5,000 NAU/kg of α-ANDV duck IgYΔFc on day 8 postinfection.

Work with the human α-ANDV FFP demonstrated that it was feasible to use polyclonal α-ANDV antibodies at doses as low as 5,000 NAU/kg to protect against lethal disease, and higher doses could be used to confer protection at later timepoints postexposure. If a polyclonal α-ANDV product could be manufactured, rather than obtained from consenting survivors, then this could provide a more consistent and unlimited source of postexposure prophylactic product to treat persons potentially exposed to ANDV. Towards this goal, we examined a novel approach to producing a candidate postexposure prophylactic to protect against HPS. This approach involved the production of polyclonal antibodies in the eggs of vaccinated ducks, and the purification of IgY and the truncated form of the antibody, IgYΔFc, from egg yolks. Ducks were vaccinated with the ANDV DNA vaccine, pWRG/AND-M, as described in the Methods section. Sera were screened for ANDV neutralizing antibodies by PRNT (data not shown). Eggs from ducks with high serum neutralizing antibody levels were collected. Ig was purified from egg yolks and samples were visualized by Western blot. Under nonreducing conditions using an α-duck IgY antibody, it is possible to visualize the IgY band, in addition to the truncated form IgYΔFc and heavy chain (HC) (**Fig. 4A**). Using signal intensity software, it was determined that the IgYΔFc and HC bands composed approximately 75% of the protein visualized by Western blot. Subsequently, the ability of purified α-ANDV duck IgY/IgYΔFc to neutralize ANDV was evaluated *in vitro* using the PRNT assay. The α-ANDV duck IgY/IgYΔFc had potent neutralizing activity, with PRNT_80_ titers of 10240, which was the same α -ANDV PRNT_80_ titer in human convalescent FFP (**Fig. 4B**).

As with α-ANDV FFP, the bioavailability of duck α-ANDV IgY/IgYΔFc was assessed in the hamster. Three hamsters each were injected with 12,000 NAU/kg or 64,000 NAU/kg of α-ANDV duck IgY/IgYΔFc by the s.c. route. Serum samples were collected on days 1, 3, 10, 15, and 21, and PRNT_80_ titers were determined. PRNT_80_ titers from hamsters administered 64,000 NAU/kg had similar levels to α-ANDV FFP on day 1; however, these titers dropped below the limit of detection following day 3. Plotting PRNT_50_ titers shows detectable titers to 6 days, with half-lives calculated to be 2.9 days for PRNT_80_ titers and 2.3 days for PRNT_50_ titers. Low neutralization titers were found after administration of 12,000 NAU/kg duck α-ANDV IgY/IgYΔFc on days 1 and 3, which then dropped below the level of detection of the assay on subsequent days (**Fig. 4C**). Half-lives were 3.0 days based on PRNT_80_ titers and 2.9 days based on PRNT_50_ titers.

Because 5,120 NAU/kg of α-ANDV FFP protected 100% of hamsters from lethal ANDV challenge, approximately the same concentration of purified α-ANDV duck IgY/IgYΔFc was tested for a capacity to protect in the HPS model. Hamsters were challenged with 4,000 PFU of ANDV i.n. on day 0 and then administered 5,000 NAU/kg of duck α-ANDV IgY/IgYΔFc (Lot #1) on day 8 postchallenge (**Table 1**). Unexpectedly, this concentration did not have a significant effect on the outcome of ANDV challenge (**Fig. 4D**). This indicated that either purified duck α-ANDV IgY/IgYΔFc was not capable of neutralizing ANDV *in vivo*, or the dosage of purified α-ANDV duck IgY/IgYΔFc required for protection was higher than 5,000 NAU/kg.

**Figure 5:**
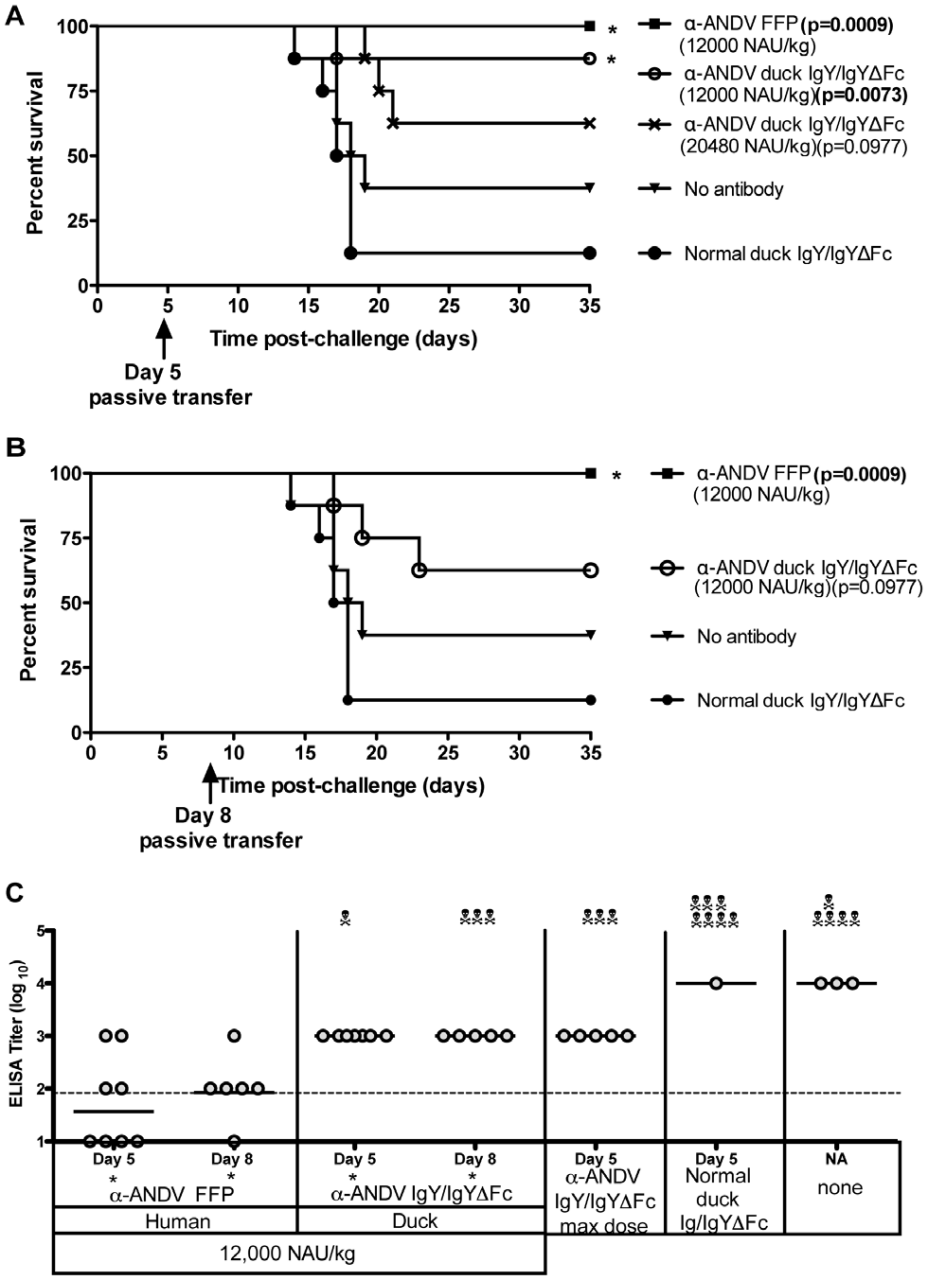
12,000 NAU/kg of α-ANDV FFP and α-ANDV duck IgY/IgYΔFc protects hamsters from lethal HPS disease. A) and B) Survival curve of hamsters that were challenged with 4,000 PFU i.n. of ANDV on day 0 and passively transferred with α-ANDV FFP or α-ANDV duck IgY/IgYΔFc on day 5 postinfection (A) or day 8 postinfection (B). * indicates statistical significance when compared to normal IgY/IgYΔFc treatment. C) α-N ELISA endpoint titers (log_10_) were conducted with sera from surviving hamsters challenged with ANDV in A) and B). GMT for each group are shown. * indicates results are statistically significant when compared to no antibody controls.

To test the possibility that a higher dose of α-ANDV IgY/IgYΔFc might confer protection, groups of 8 hamsters were challenged with 4,000 PFU i.n. of ANDV on day 0, and were then administered duck α-ANDV IgY/IgYΔFc (Lot #2) at two concentrations, 12,000 and 20,480 NAU/kg, on either day 5 or 8 post-challenge (**Table 1**). α-ANDV FFP at 12,000 NAU/kg was included as a positive control. Purified normal duck IgY and untreated hamsters were included as negative controls. As expected, α-ANDV FFP administered on day 5 elicited complete protection. Hamsters receiving 12,000 NAU/kg of α-ANDV duck IgY/IgYΔFc on day 5 were also significantly protected (88% survival, *P* = 0.0073). Hamsters receiving 20,480 NAU/kg of α-ANDV duck IgY/IgYΔFc had decreased survival (63% survival, *P* = 0.0977); which is not statistically significant when compared to normal duck IgY. (**Fig. 5A**). When 12,000 NAU/kg of either α-ANDV FFP or duck α-ANDV IgY/IgYΔFc (Lot #2) were administered 8 days post-ANDV challenge, α-ANDV FFP completely protected hamsters (100% survival, *P* = 0.0009); whereas duck α-ANDV IgY/IgYΔFc exhibited incomplete protection (63% survival, *P* = 0.0977) (**Fig. 5B**) that was not statistically significant compared to negative controls.

To determine if hamsters had been productively infected, sera from surviving hamsters were subjected to a nucleocapsid ELISA. Hamsters receiving α-ANDV FFP on days 5 or 8 had lower levels of α-nucleocapsid antibodies when compared to hamsters receiving the same 12,000 NAU/kg dose of duck α-ANDV IgY/IgYΔFc (**Fig. 5C**) suggesting that human FFP was more effective at limiting infection than the duck IgY/IgYΔFc having the same in vitro neutralizing activity. When compared to no antibody controls, the reduction of the FFP ELISA titer was statistically significant (*P* = 0.0148 on day 5 and *P* = 0.0097 on day 8) while the duck IgY/IgYΔFc was also statistically significant (*P* = 0.0043 on day 5 and *P* = 0.0135 on day 8). Taken together, 12,000 NAU/kg of duck α-ANDV IgY/IgYΔFc administered 5 or 8 days after .i.n. exposure limited infection but only the treatment on day 5 was effective at conferring a significant level of protection against lethal disease.

### Direct comparison of α-ANDV FFP and duck α-ANDV IgY/IgYΔFc for a capacity to protect hamsters from lethal ANDV i.m. challenge

**Figure 6:**
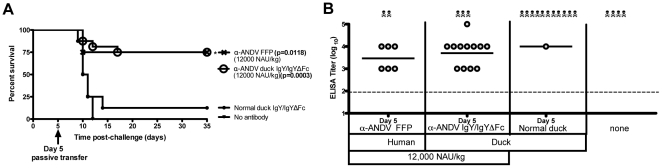
12,000 NAU/kg of anti-ANDV duck IgY/IgYΔFc protects hamsters from lethal HPS disease. A) Survival curve of hamsters that were challenged with 200 PFU i.m. of ANDV on day 0 and passively transferred with α-ANDV FFP (8 hamsters per group) or α-ANDV duck IgY/IgYΔFc (16 hamsters per group) on day 5 postinfection. * indicates statistical significance when compared to normal IgY/IgYΔFc treatment. B) α-N ELISA endpoint titers (log_10_) were conducted with sera from surviving hamsters challenged with ANDV in A). GMT for each group are shown.

Having found that the manufactured α-ANDV IgY/IgYΔFc was capable of conferring protection in the i.n. model, we were interested in testing whether this material could protect in a more aggressive i.m. challenge model. When Syrian hamsters were challenged i.m. with 200 PFU of ANDV the mean day-to-death was 11, which was 7 days earlier than the mean day-to-death in the i.n. model [16]. Groups of 8 to 16 hamsters were challenged with 200 pfu of ANDV by the i.m. route and then, 5 days after exposure, the hamsters were treated with 12,000 NAU/kg of either α-ANDV FFP or α-ANDV IgY/IgYΔFc. Both α-ANDV FFP and α-ANDV duck IgY/IgYΔFc conferred protection to 75% of the hamsters in each group (FFP n = 8, duck IgYΔFc n = 16) (**Fig. 6A**). Protection was statistically significant when compared to hamsters receiving nonspecific duck IgY with *P* values of 0.0118 and 0.0003, respectively.

To determine if surviving hamsters had been productively infected, sera from surviving hamsters were subjected to nucleocapsid ELISA. All surviving hamsters receiving either α-ANDV FFP or α-ANDV duck IgY/IgYΔFc mounted an immune response to ANDV indicating all animals were productively infected, but nevertheless protected from lethal disease (**Fig. 6B**). These results demonstrate α-ANDV FFP and α-ANDV duck IgY/IgYΔFc are capable of protecting from lethal ANDV infection by both the respiratory and intramuscular challenge routes when administered 5 days postinfection.

## Discussion

Previous passive transfer studies using the ANDV/hamster model revealed that it was possible to use a postexposure prophylactic to protect against HPS [14], [16]. This experimental work, coupled with epidemiologic studies demonstrating that ANDV was associated with person-to-person transmission [5], indicated that it should be possible to use α-ANDV neutralizing antibodies to treat and/or prevent disease among close contacts of HPS cases. This would be done by administering α-ANDV neutralizing antibodies before contacts of HPS cases show any signs of disease. A clinical trial addressing this possibility using α-ANDV FFP is ongoing in Chile (unpublished data). If α-ANDV FFP shows promise in the clinical trials, the issue of how to obtain sufficient quantities of immune plasma for clinical use remains a challenge. The necessity of using blood group-typed immune plasma for patients increases the complexity of using α-ANDV FFP. Like many zoonotics, HPS can be unpredictable in a natural setting and therefore, estimating the location, timing, and magnitude of outbreaks is difficult. Moreover, ANDV is a category A agent on the NIAID list of potential biological threat agents. In the event of a large-scale outbreak, the emergency use of FFP collected from consenting HPS survivors would likely be inadequate as a response or deterrent. Other approaches that have been used to produce immunotherapeutics to viral diseases include collecting sera from persons vaccinated with a licensed vaccine (examples include hepatitis B virus, cytomegalovirus, vaccinia virus, and rabies virus [29]) or developing protective monoclonal antibodies (e.g., Synagis for treatment of respiratory syncytial virus). Unfortunately, there are no ANDV vaccines in advanced development, and there are no ANDV neutralizing/protective monoclonal antibodies identified. An alternative approach to develop an α-ANDV immunotherapeutic is to produce polyclonal antibodies in animals using processes used routinely to produce α-venoms and α-toxins.

There are several FDA-approved animal-derived polyclonal immunoglobulin products. Most of these are α-venoms or α-toxins made in horses or sheep vaccinated with purified detoxified venoms or toxins obtained from their natural source. For most of the modern products, enzymatic treatment of the equine or ovine antibody is performed to “despeciate" the antibody by removing the heterologous Fc fragment. For example, the FDA recently approved Anascorp, a papain-treated polyclonal antibody F(ab′)_2_ against scorpion toxin produced in horses [14], [16]. Papain is a known human allergen, sharing similar antigenic structures with some dust mite and latex allergens, further limiting its usage for patients with those known allergies [30], [31]. This despeciation process, enzymatically separating the Fab′ fragments from the Fc, reduces the possibility of a hypersensitivity reaction to the Fc fragment, but may retain risk associated with enzyme hypersensitivity. Several years ago, this polyclonal antibody approach was proposed as a means to defend against viruses and other agents used as biological weapons [32]. However, it is notable that there are no licensed animal-derived polyclonal immunoglobulin products against any viral disease.

Proof-of-concept studies where chickens or, more recently, ducks were vaccinated with various venoms, toxins, or infectious agents to produce antibody that could be purified from egg yolks have been reported [33], [34], [35]. In most cases, the vaccine antigens were purified proteins; however, there were reports of the use of DNA vaccines to produce the IgYΔFc in ducks [24], [25], [36]. Ours is the first report describing the use of DNA vaccine technology to produce antiviral polyclonal antibody in duck eggs that were then tested *in vitro* and then *in vivo* as a postexposure prophylactic. This same DNA vaccine technology could be used to vaccinate equine or ovine species to develop α-ANDV polyclonal antibodies.

Reconstituted lyophilized plasma from HPS survivors had previously been shown to protect in the ANDV/hamster lethal HPS model [14]. In that study, a dose of approximately 50,000 NAU/kg injected i.p. protected 50% of the hamsters challenged i.m. with 250 LD_50_ of ANDV. Here, hamsters given at least 5,000 NAU/kg of α-ANDV FFP s.c. as late as 8 days post-ANDV i.n. challenge were uniformly protected against lethal HPS disease. Doses of FFP below 5,000 NAU/kg did not confer protection. Similarly, treatment after day 8 did not confer significant protection against an i.n. challenge (**Fig. 2** and **Fig. 3**). These data confirmed that it is possible to use human convalescent plasma to protect against HPS and continued to refine the protective dose and treatment window. In general, the data indicate that a higher dosage of FFP is required to confer protection as more time elapses after exposure, and that the treatment window appears to close between 5–9 days before the mean day-to-death for an i.n. challenge (i.e., day 17). Note that all of the passive transfer experiments described in this report involve a single injection of antibody. An actual treatment regime in the clinic would likely involve multiple dosing to achieve and maintain a high level of neutralizing activity over time. This would become of greater importance since the half-life of α-ANDV IgY/IgYΔFc is approximately half of α-ANDV FFP. Such a continuous treatment regimen also highlights the importance of having limited reactogenic material, which duck antibodies lacking the Fc regions is expected to possess.

A notable finding in this study was that a manufactured product, α-ANDV IgY/IgYΔFc, could protect against lethal HPS to levels similar to human FFP. A dose of 12,000 NAU/kg IgY/IgYΔFc delivered five days postexposure conferred significant protection in both the i.n. and i.m. challenge models (**Fig. 5A** and **6A**). To our knowledge, this is the first time an antiviral biologic produced in duck eggs has been used as a postexposure prophylactic to protect against a lethal viral disease. The protection was dependent on the day of delivery because 12,000 NAU/kg of IgY/IgYΔFc delivered on day 8 was not protective (**Fig. 4D** and **5B**). This finding indicated that the FFP was more effective at conferring *in vivo* protection than IgY/IgYΔFc because a lower dose of 5,000 of FFP was capable of protecting when delivered on day 8 (**Fig. 3**). FFP consists primarily of α-ANDV immunoglobulin with the Fc portion of the antibody intact. This Fc could be involved in mechanisms of complement dependent and independent protection not measured in the PRNT including downstream signaling to other immune cells to sites of bound antigen, release of inflammatory mediators, endocytosis, and phagocytosis. In contrast, it is likely that the protection conferred by the IgY/IgYΔFc involves only the virus-binding region of the antibody and not any additional effector functions. This is because the majority of the Fc region is naturally absent and what remains is likely incompatible with the mammalian Fc receptors [37]. Although this Fc- property likely reduces the antiviral potency of the product, it also likely reduces the reactogenicity of the product. This is because the Fc region of heterologous antibodies plays a dominant role in cross-species reactogenicity [24]. Further testing will be required to determine if the duck IgY/IgYΔFc is less reactogenic than Ig or despeciated Ig produced in equine or ovine derived polyclonal antibodies.

Nucleocapsid ELISAs conducted with serum from surviving hamsters shows that hamsters passively transferred with the higher doses of α-ANDV FFP on days 5 or 8 had low or undetectable levels of α-nucleocapsid antibodies 1 month after challenge. This was unexpected because 250 LD_50_ of ANDV had been *in vivo* for several days before treatment and it seemed probable that enough replication would have occurred elicit a robust α-N response. The low α-N titers in the hamsters administered high doses of neutralizing antibodies after i.n. exposure indicates that ANDV does not replicate at high levels and disseminate immediately after intranasal infection. This argument is supported by a recent publication by Safronetz, et. al., stating that Andes virus RNA is not detected in the blood until a few days before the animals succumb following intranasal challenge. Also, the authors show a suppression of the early innate immune response in most organs [38]. Therefore, a high dose of neutralizing antibodies administered to hamsters within 8 days after exposure to ANDV apparently is sufficient to contain and suppress the infection. In contrast, if the neutralizing antibody dosage is lower as in the case of the α-ANDV rabbit sera (**Fig. 2**), then the treatment is capable of protecting the hamsters against lethal disease, but not against a level of ANDV amplification sufficient to elicit a robust α-N antibody response.

A major implication of this work is that duck antibody can function as an α-ANDV postexposure prophylactic. To be practical, this would require scaling up production to meet demand. We demonstrated that 5,000 NAU/kg of α-ANDV FFP was able to protect 100% of hamsters from lethal HPS disease if administered as late as 8 days after exposure. When scaled up for a 70 kg human, a dose would be equivalent to 34 ml of α-ANDV FFP, with 13 doses per unit of blood. A more conservative approach would be to administer 12,000 NAU/kg of α-ANDV FFP, which not only protected against lethal disease, but also limited the infection as measured by seroconversion. A 12,000 NAU/kg dose corresponds to 82 ml of α-ANDV FFP or five doses per unit of blood. 12,000 NAU/kg of α-ANDV duck IgY/IgYΔFc was protective if administered within 5 days of exposure in the ANDV/hamster model. Scaling this up for a 70 kg human, would give a dose equal to 82 ml of purified α-ANDV duck IgY/IgYΔFc, at a concentration of 910 mg/dose. Duck egg yolks contain only the IgY class of antibodies [24], [37], with a potential yield of 100 mg of purified IgY from each egg yolk [25], [39]. By vaccinating ducks with the pWRG/AND-M DNA vaccine, hyperimmune egg yolks containing IgYΔFc can be manufactured on a larger scale, without the need to bleed the duck. Based on the ∼1,000 mg dose, 10 eggs would be required per dose. Using this rough estimate, 1,000 human doses would require 10,000 eggs. Ducks lay approximately 5 eggs per week; therefore, it would take only 200 vaccinated ducks 10 weeks to produce 1,000 human doses.

Postexposure prophylaxis of suspected rabies virus infection involves vaccination along with immunoglobulin treatment. Passive transfer of anti-rabies immunoglobulin introduces neutralizing antibodies that are immediately able to combat the virus, while the host is generating its own antibodies from the viral antigens introduced through administration of the rabies vaccine. A similar strategy could be employed for hantavirus infection. Co-administering both the ANDV DNA vaccine and antibody therapy postexposure would allow antibodies to neutralize virus immediately while the host generates virus-specific antibodies from vaccination. The use of a DNA vaccine, rather than an attenuated virus vaccine, might be preferable because passively transferred neutralizing antibodies would not inhibit the host response to vaccination. In previous experiments, we observed that hamsters treated with neutralizing antibodies from DNA-vaccinated monkey immune serum before ANDV exposure were either completely protected against infection, or exhibited a significant delay in death (i.e., mean day-to-death of 42 days versus 11 days for hamsters treated with normal monkey sera) [14]. We hypothesized that the delay in the disease course is due to incomplete neutralization of virus after treatment with immune sera. As the host clears the heterologous antibodies, the virus that escaped neutralization by passive transfer is able to amplify, disseminate, and eventually cause lethal HPS in the host. DNA vaccination concurrent with antibody therapy could effectively prevent this from occurring by eliciting an active immune response that would eliminate virus that had escaped the initial passive transfer of neutralizing antibodies.

### Summary

Together, these experiments demonstrated that currently available convalescent FFP can be used as a postexposure prophylactic, and importantly, demonstrated that it is feasible to use DNA vaccine technology coupled with the duck/egg system to manufacture postexposure immunoprophylactics to prevent HPS. The DNA vaccine-duck/egg system can be scaled as needed and obviates the necessity of using limited blood products obtained from a small number of HPS survivors. This is the first report demonstrating the *in vivo* efficacy of any antiviral product produced using the DNA vaccine-duck/egg system. Whether this system will be viable as a means to develop products that are practical, safe, and effective in humans will require further evaluation.
